# Association between asthma and type 2 diabetes in a Swedish adult population: a register-based cross-sectional study

**DOI:** 10.1136/thorax-2024-222819

**Published:** 2025-03-23

**Authors:** Mwenya Mubanga, Tong Gong, Awad I Smew, Amanda Wikström, Emma Caffrey Osvald, Katarina Eeg-Olofsson, Christer Janson, Cecilia Lundholm, Catarina Almqvist

**Affiliations:** 1 Department of Medical Epidemiology and Biostatistics, Karolinska Institutet, Stockholm, Sweden; 2 HIV Surveillance Unit, Center for Infectious Disease Research, Lusaka, Zambia; 3 Pediatric Allergy and Pulmonology Unit, Astrid Lindgren Children’s Hospital, Stockholm, Sweden; 4 Västra Götalandsregionen, Vanersborg, Sweden; 5 Department of Molecular and Clinical Medicine, University of Gothenburg, Goteborg, Sweden; 6 Department of Medical Sciences, Respiratory Medicine, Uppsala University, Uppsala, Sweden

**Keywords:** Asthma, Asthma Epidemiology

## Abstract

**Objective:**

Asthma and type 2 diabetes are two important causes of morbidity globally. We examined both the association of type 2 diabetes with asthma in Swedish adults and the familial co-aggregation of the diseases.

**Methods:**

We conducted a cross-sectional study of all adults aged 25–85 in Sweden between 2009 and 2013. Asthma and type 2 diabetes status were ascertained from the health registers. Models were adjusted for sex, age, education level, income and country of birth and in a subset, for body mass index (BMI). We further conducted a familial coaggregation analysis to determine if shared familial factors could explain any observed findings.

**Results:**

The study included 5 299 245 participants, 25 292 (0.5%) had both asthma and type 2 diabetes. In the total population, the OR for the association between type 2 diabetes and asthma was 1.47 (95% CI 1.45 to 1.49); in the population of men (1.30 (95% CI 1.27 to 1.32)) and women (1.63 (95% CI 1.60 to 1.66)). The ORs were slightly higher among men (1.51 (95% CI 1.45 to 1.56)) and women (2.04 (95% CI 1.96 to 2.11)) for whom BMI measurements were available but attenuated with adjustment for BMI (1.45 (95% CI 1.40 to 1.51)) and (1.76 (95% CI 1.68 to 1.84)). Diabetes was more likely if a full sibling had asthma than if the sibling did not (1.13 (95% CI 1.10 to 1.15)).

**Conclusions:**

We found an association between asthma and type 2 diabetes that was sustained after adjusting for BMI, indicating that BMI alone does not explain this relationship. We also found that the two conditions coaggregate in siblings, indicating that the association is partly due to shared familial genetic and environmental risk factors.

**WHAT IS ALREADY KNOWN ON THIS TOPIC**

- There are conflicting findings regarding the within-person association between asthma and type 2 diabetes, but little is known about the possibility of familial coaggregation of both diseases.

**WHAT THIS STUDY ADDS**

- Shared familial risk factors explain some of the association between type 2 diabetes and asthma.
- BMI is an important confounder in the within-person association between asthma and type 2 diabetes.

**HOW THIS STUDY MIGHT AFFECT RESEARCH, PRACTICE OR POLICY**

- There needs to be an increased index of suspicion for asthma and type 2 diabetes in family members.

## Introduction

 Asthma and type 2 diabetes are two complex and prevalent diseases, each constituting a significant public health problem. Studies have shown that both may coexist in the same individual,^1^,^3^ and it is hypothesised that this may be due to several possible mechanisms. Both conditions share in common a sustained proinflammtory state,^4 5^ which may or may not be associated with obesity.^2 6 7^ Asthma is associated with the upregulation of multiple proinflammatory cytokines, possibly through the activation of nuclear factor (NF)-κB, such as interleukin (IL)-4, IL-5, IL-6, IL-13 and IL-17, tumour necrosis factor (TNF)-α and different adhesion molecules.^8 9^ It has been proposed that constantly raised levels of these cytokines may cause insulin resistance, drive systemic inflammation and consequently result in type 2 diabetes.^2 5 10 11^ There is also evidence that asthma is a systemic disorder with significant cross-communication through inflammatory mediators to other distant organs.^8 9^ Further, hormone imbalances as observed in hyperleptinaemia, leptin resistance and hyperinsulinaemia are important in the development of both asthma and type 2 diabetes.^3 12 13^ Insulin plays an important role in physiological lung development, supporting alveolarisation. In hyperinsulinaemia, the levels interfere with lung development and maturation, while facilitating a proasthmatic immune environment.^14 15^

The underlying factors associated with asthma, and independently with type 2 diabetes, are known to be dependent on several risk factors,^8 10^ with the phenotypic expression being modified by genetic and environmental factors.^1 16^ An important common risk factor for both diseases is obesity. Evidence exists for the shared genetics between asthma and obesity,^17^ and similarly, type 2 diabetes is a trait strongly correlated with obesity.^16^ While previous studies have shown conflicting findings regarding the within-person relationship between asthma and type 2 diabetes, to the best of our knowledge, there have been very few studies that have attempted to study the possibility of familial coaggregation of both diseases.

Familial coaggregation studies offer evidence for familial clustering of two diseases, allowing investigators to test for the presence of shared genetic and/or environmental factors that may contribute to this association.^18^ To further advance the understanding of the relationship between asthma and type 2 diabetes in the Swedish population, we carried out a population-based cross-sectional study using the rich Swedish national registers, with the aim of examining both the association of type 2 diabetes with asthma in adults in this population regarding the familial coaggregation of the diseases.

## Methods

### Study design

We conducted a register-based cross-sectional study with prospectively recorded data that included all Swedish singleton adults born between 1924 and 1984 (n=8 163 216). All participants had to be alive and residents of Sweden for the full duration of the study between January 2009 and December 2013.

Participants were identified using the Register of the Total Population with information on birth, migration and civil status of all Swedish residents.^19^ From the population of 8 163 216, we excluded people who died before 1 January 2014 (n=1 138 473), those who had not resided in Sweden during the full duration of the study (n=432 199) to ensure complete coverage of diagnoses and medication in the registers and those who emigrated from Sweden (1 045 242). It was possible to extract information on physician-diagnosed asthma or type 2 diabetes from the National Patient Register^20^ and the Swedish Prescribed Drug Register.^21^ The National Patient Register has had complete coverage for inpatient hospital diagnoses since 1987, and approximately 80% of outpatient specialist care visits since 2001. The Swedish Prescribed Drug Register has been available since July 2005 and contains information on drugs indicated for treatment which have been dispensed at pharmacies in Sweden.

Further, all participants who had a diagnosis of type 1 diabetes in the National Patient Register or were dispensed relevant medication from the Swedish Prescribed Register were also excluded. Type 1 diabetes was defined as the International Classification of Diseases (ICD)-10 E10 and/or insulin or insulin analogues (Anatomical Therapeutic Chemical (ATC) A10A), without any other blood glucose-lowering drugs ever^22^ (n=85 213).

Due to the overlap in medication for asthma and chronic obstructive pulmonary disease (COPD), we additionally excluded 172 917 participants with COPD in either the National Patient Register (ICD-10 J44) or the Swedish Prescribed Drug Register (ATC codes R03AL (beta-2 agonists in combination with anticholinergics including triple combinations with corticosteroids) and R03BB (anticholinergics)). Finally, we also excluded everyone missing socioeconomic information from the registers (n=59 927).

**Figure 1:**
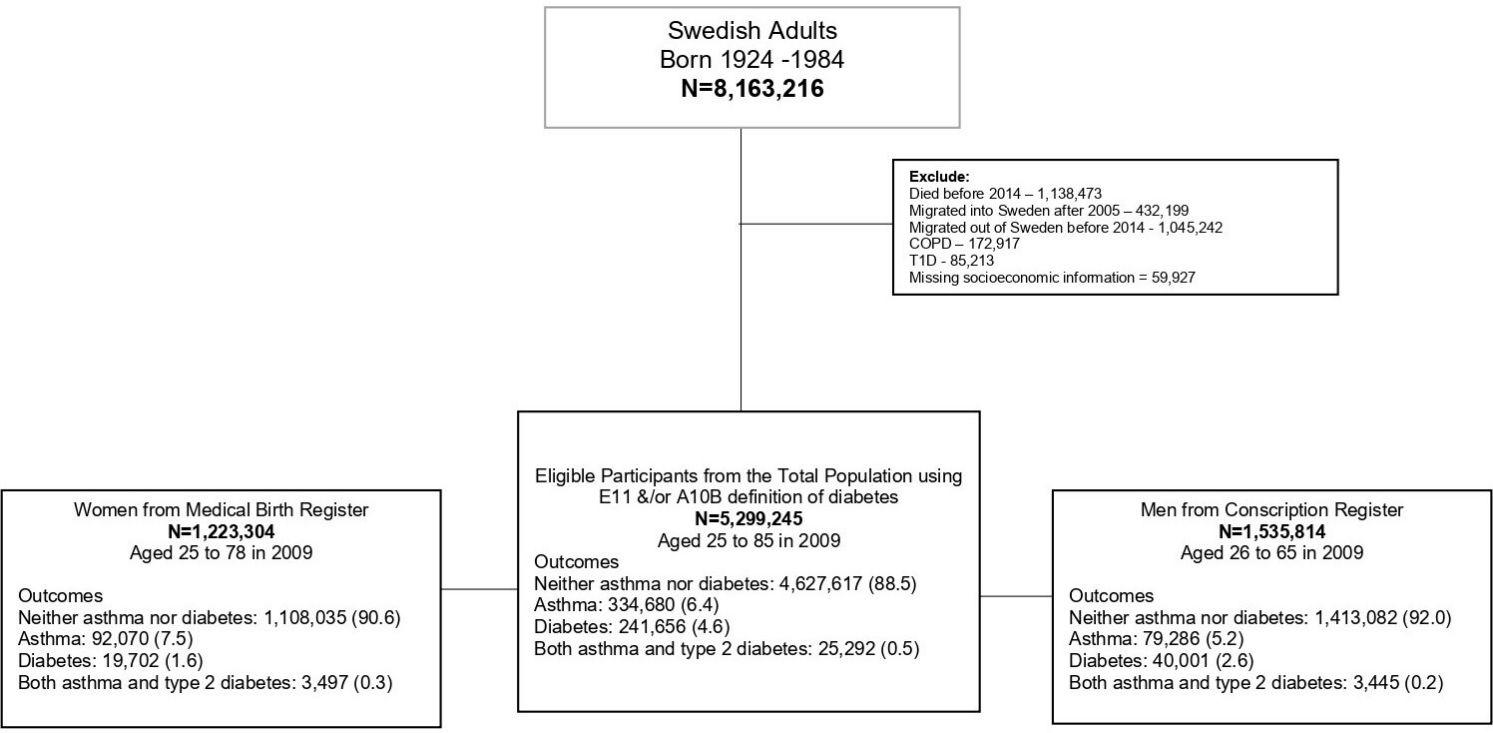
Eligibility flow chart. COPD, chronic obstructive pulmonary disease; T1D, type 1 diabetes.

We further distinguished between type 2 diabetes as defined by ICD-10 E11, and other forms of diabetes. These ‘other’ forms include malnutrition-related diabetes, gestational diabetes, unspecified diabetes or anyone on mixed insulin and oral hypoglycaemic medications. The other forms of diabetes were included under an extended diabetes definition. Of the 288 573 participants found with diabetes, 21 625 were identified with other forms of diabetes (extended diabetes). The exclusion left a final population of 5 229 245 (figure 1).

Information on family relations (full siblings) was further identified from the Multi-Generation Register.^23^ First, both biological parents’ personal identity numbers were used as the nuclear family ID to identify all sibling pairs. For families with more than two siblings, we have generated all possible sibling pairs for comparison (eg, six pairs for a three-sibling family who share the same family ID would be identified: sib1-sib2, sib1-sib3, sib2-sib3, sib3-sib2, sib3-sib1 and sib2-sib1). The pairs of full siblings within the study population who fell into the age category 45–78 in 2009 were then selected and included in the family coggregation analyses (n=3 153 219), out of whom 750 153 were full brothers and 822 241 were full sisters.

Body mass index (BMI) was only available for a subset of the population. It was possible to retrieve information on BMI for a subset of men from the Swedish Conscription Register enlisted between 1967 and 2001 (n=1 535 814; 29.0% of the total population). This register includes information on all military conscripts around 18 years of age.^24^ We then extracted information on BMI for pregnant women in the Medical Birth Register (n=1 223 304; 23.1% of the total population). The register reports >98% of all births in Sweden annually (established 1973) and includes maternal information such as BMI in early pregnancy at the first antenatal booking from 1982 and onwards^25^ (figure 1).

The Longitudinal Integration Database for Health Insurance and Labor Market Studies includes microdata about highest attained education and income by calendar year.^26^ We used it to extract information on socioeconomic status measured as education and household disposable income in 2011. Further information regarding the registers is included in the online supplemental material.

### Dependent variable

Type 2 diabetes was defined as either physician-diagnosed in the National Patient Register^20^ ICD-10 code E11 (n=155 396) and/or as blood glucose-lowering drugs except insulin (ATC-code A10B) in the Swedish Prescribed Drug Register (n=263 686). All cases had to fulfil the definition of current disease at any time between 2009 and 2013.

For additional analysis, we also identified all the other types of diabetes mellitus (extended diabetes definition) based on ICD-10 E11 in addition to the other non-insulin dependent definitions of diabetes including gestational diabetes O24 (3442), malnutrition-related diabetes mellitus ICD-10 E12 (n=126), other specified diabetes mellitus ICD-10 E13 (n=795) and unspecified diabetes mellitus ICD-10 E14 (n=17 424, online supplemental table 1).

To compare the association between asthma and diabetes using the different drug groups used to treat type 2 diabetes; we further identified biguanides (ATC A10BA; n=248 029), sulfonylureas (ATC A10BB; n=71 821), thiazolidinediones (ATC A10BG; n=7915) and combined oral hypoglycaemic use (ATC A10BD; n=6113).

### Independent variable

Asthma was identified in the National Patient Register as ICD-10 J45 or J46 and/or identified in the Swedish Prescribed Drug Register as inhalations of selective β2-adrenoreceptor agonists (ATC R03AC), glucocorticoids (R03BA), fixed combinations of β2-agonists and glucocorticoids (R03AK) and leukotriene receptor antagonists (R03DC) according to a previously validated algorithm (n=359 972).^27^ It was not possible to determine which individuals had previously been diagnosed with paediatric asthma as the Swedish Prescribed Drug Register has only been in existence since 2004 and would not cover childhood for all participants.

### Covariates

**Figure 2:**
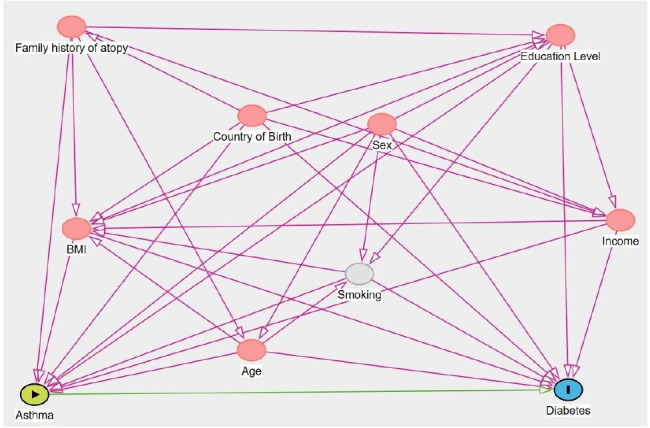
Directed acyclic graph. BMI, body mass index.

Covariates were selected based on a directed acyclic graph (figure 2). Variables identified included age (continuous variable), sex (male/female), education level (primary ≤9 years, secondary 10–12 years, tertiary >12 years) and country of birth (Sweden, other Nordic country, non-Nordic country). Income was categorised into quintiles by birth year (1=lowest, 5=highest). This variable was created by dividing the annual household income into five groups, divided by quintiles, from lowest income to highest for each birth year. BMI was categorised as underweight (<18.5 kg/m^2^), healthy weight (18.5–24.9 kg/m^2^), overweight (25.0–29.9 kg/m^2^) and obesity (≥30 kg/m^2^)).

### Statistical analyses

Logistic regression was used to estimate ORs with 95% CIs examining the association between type 2 diabetes (dependent variable) and asthma (independent variable) with cluster-robust sandwich estimator to account for dependencies between siblings. In the first part of the study, this was done to measure within-person association in the total population, in only the men and then only in the women. Adjustment was made in three steps in the total population. First, we adjusted for age (continuous); then age and sex; then fully adjusted for age, sex, education level, income and birth country. We then repeated all three steps in a subset of the men only and women only populations, additionally adjusting for BMI.

In the second part of the study, we estimated the familial coaggregation of type 2 diabetes (dependent) and asthma (independent). Using logistic regression with cluster-robust sandwich estimator to account for dependencies between sibling pairs, we estimated ORs and 95% CIs of type 2 diabetes in siblings of index persons with asthma compared with siblings of index persons without asthma. We adjusted for sex, age, siblings’ asthma, income and education level. The analysis was done in the population of full siblings, among pairs of brothers, pairs of sisters and pairs of siblings of the opposite sex. If an index case had more than one eligible sibling, these were analysed as separate siblings’ pairs.

In a sensitivity analysis, we repeated the analysis estimating the association between an extended definition of diabetes (dependent, see exclusion above) and asthma (independent). We also estimated the association between asthma and diabetes treated with oral hypoglycaemic drugs (biguanides, sulfonylureas, thiazolidinediones and mixed oral hypoglycaemic drug use).

All analyses were conducted using Stata V.16.

## Results

Out of 5 229 245 Swedish residents, 2 577 583 (49.3%) were male, 2 086 792 (39.9 %) were aged between 45 and 65, and 4 527 082 (86.6%) were born in Sweden. Additionally, 334 680 (6.4%) of the adults had asthma only, 241 656 (4.6%) had type 2 diabetes only and 25 292 (0.5%) had both asthma and type 2 diabetes. The baseline features of the study participants are detailed in table 1. We additionally show the baseline characteristics of the total population included as having the extended diabetes diagnosis in online supplemental table 2. Similar to the main study population, out of 5 250 870 Swedish residents, 2 588 027 (49.3%) were male, 2 094 413 (39.9 %) were aged between 45 and 65, and 4 544 298 (86.5%) were born in Sweden. In the characteristics of the men only and women only populations in online supplemental table 3; of 1 535 816 men, 1 128 077 (73.4%) had a BMI of 18.5–24.9 and most aged <45 years (n=950 827; 61.7%); while from the women only cohort, there were 1 222 304 women with 722 852 (59.1%) of these categorised with a BMI of 18.5–24.9 kg/m^2^ and most aged <45 (n=708 446; 57.9%).

**Table 1:** Baseline characteristics*

	Total population	No asthma or diabetes	Asthma only	Diabetes only	Both asthma and diabetes
Characteristics					
Number (%)	5 229 245 (100)	4 627 617 (88.5)	334 680 (6.4)	241 656 (4.6)	25 292 (0.5)
Sex					
Male	2 577 583 (49.3)	2 293 656 (49.6)	130 918 (39.1)	142 226 (58.8)	10 783 (42.6)
Age category					
<45	2 140 963 (40.9)	1 986 918 (42.9)	130 987 (39.1)	20 332 (8.4)	2726 (10.8)
45–65	2 086 792 (39.9)	1 821 035 (39.4)	138 491 (41.4)	114 709 (47.5)	12 557 (49.6)
>65	1 001 490 (19.2)	819 664 (17.7)	65 202 (19.5)	106 615 (44.1)	10 009 (39.6)
Education level					
Compulsory	1 048 647 (20.1)	887 141 (19.2)	62 928 (18.8)	90 116 (37.3)	8462 (33.5)
Secondary	2 358 918 (45.1)	2 092 335 (45.2)	150 927 (45.1)	104 211 (43.1)	11 445 (45.2)
Tertiary	1 794 295 (34.3)	1 625 178 (35.1)	119 513 (35.7)	44 513 (18.4)	5091 (20.1)
Missing	27 385 (0.5)	22 963 (0.5)	1312 (0.4)	2816 (1.2)	294 (1.2)
Quantiles of income					
1 (lowest)	1 044 425 (20.0)	913 880 (19.8)	67 523 (20.2)	56 395 (23.2)	6627 (26.2)
2	1 045 588 (20.0)	918 353 (19.8)	70 433 (21.0)	50 948 (21.1)	5854 (23.1)
3	1 045 746 (20.0)	923 701 (20.0)	67 822 (20.3)	49 201 (20.4)	5022 (19.9)
4	1 046 396 (20.0)	931 525 (20.1)	64 108 (19.1)	46 479 (19.2)	4284 (16.9)
5 (highest)	1 047 090 (20.0)	940 158 (20.3)	64 794 (19.4)	38 633 (16.0)	3505 (13.9)
Country of birth					
Sweden	4 527 082 (86.6)	4 016 345 (86.8)	293 392 (87.7)	196 957 (81.5)	20 388 (80.6)
Other Nordic	161 453 (3.1)	137 249 (3.0)	11 973 (3.6)	10 911 (4.5)	1320 (5.2)
Other countries	540 562 (10.3)	473 887 (10.2)	29 308 (8.8)	33 784 (14.0)	3583 (14.2)
Missing	148 (<0.0)	136 (<0.0)	7 (<0.0)	4 (<0.0)	1 (<0.0)

* Describes the characteristics for the population of 5 229 245 between 2009 and 2013. Education level and quantiles of income are based on LISA data from 2011. Diabetes is defined as ICD-10 E11 and/or ATC code-A10B.

ATC, Anatomical Therapeutic Chemical; ICD-10, International Classification of Diseases, 10th Revision; LISA, Longitudinal Integration Database for Health Insurance and Labour Market Studies.

### Asthma and type 2 diabetes (ICD-10 E11 and/or ATC code A10B)

In participants with asthma, there was an increased odds of type 2 diabetes observed in the total population on adjustment for age and sex (OR 1.44; 95% CI 1.42 to 1.46); and in the fully adjusted model (OR 1.47; 95% CI 1.45 to 1.49). In men among the total population, an association was observed after age-adjusted analyses (OR 1.28; 95% CI 1.25 to 1.30), which was sustained even after full adjustment (OR 1.30; 95% CI 1.27 to 1.32). Similarly, in the women-only population, an increased odds was observed in the age-adjusted analyses (OR 1.60; 95% CI 1.57 to 1.63), which remained even on full adjustment (OR 1.63; 95% CI 1.60 to 1.66).

In the Conscription Register (subgroup of men), we observed that, like men in the total population, there was an association observed between asthma and type 2 diabetes. However, on adjustment for BMI, there was an attenuation of the observed odds; (age-adjusted OR, 1.49; 95% CI 1.44 to 1.55 and fully adjusted OR 1.45; 95% CI 1.40 to 1.51). Similarly, in the Medical Birth Register (subgroup of women), we observed that similar to the women in the total population, there was an association observed which attenuated after adjustment for BMI (age-adjusted OR 2.04; 95% CI 1.97 to 2.12 and fully adjusted OR 1.76; 95% CI 1.68 to 1.84) (table 2).

**Table 2:** ORs for the within-person association between asthma and type 2 diabetes using a type 2 diabetes definition of ICD-10 E11 and/or ATC code A10B

Population	N*	N†	Population included	Model 1OR (95% CI)	Model 2‡OR (95% CI)	Model 3‡OR (95% CI)	Model 4‡OR (95% CI)
Total population	5 229 245	5 201 728	Male and female	1.37 (1.36 to 1.39)	1.44 (1.42 to 1.46)	1.47 (1.45 to 1.49)	–
Total population(men only)	2 577 583	2 564 529	Male only	1.28 (1.25 to 1.30)	–	1.30 (1.27 to 1.32)	–
Total population(women only)	2 651 662	2 637 199	Female only	1.60 (1.57 to 1.63)	–	1.63 (1.60 to 1.66)	–
Men from Conscription Register	1 535 814	1 407 228	Male only	1.49 (1.44 to 1.55)	–	1.51 (1.45 to 1.56)	1.45 (1.40 to 1.51)
Women from Medical Birth Register	1 223 304	1 221 637	Female only	2.04 (1.97 to 2.12)	–	2.04 (1.96 to 2.11)	1.76 (1.68 to 1.84)

Model 1: age adjusted.

Model 2: adjusted for age, sex*.

Model 3: adjusted for age, sex*, education level, income, birth country.

Model 4: adjusted for age, sex*, education level, income, birth country, body mass index.

* Total number of people in the specified cohort.

† Number of participants in full model.

‡ Sex*: This covariate is not included for the men only and female only cohorts.

ATC, Anatomical Therapeutic Chemical; ICD-10, International Classification of Diseases, 10 Revision.

From the full siblings (same mother and father) identified in the Multi-Generational Register (n=3 153 219), 750 153 were brothers and 822 241 sisters. Among the full siblings of individuals with one disease, the prevalence of the other disease was higher. In index participants who had asthma, the adjusted OR of type 2 diabetes in their relatives was 1.13 (95% CI 1.10 to 1.15) compared with index persons without asthma (table 3). Furthermore, full brothers and full sisters with asthma were at increased odds of type 2 diabetes compared with the relatives of individuals without other disease seen in fully-adjusted models (OR 1.12; 95% CI 1.08 to 1.16 and OR 1.13; 95% CI 1.10 to 1.17, respectively).

**Table 3:** ORs for type 2 diabetes in relatives of individuals with and without asthma

Type of relative	Unique index individuals	Model 1	Model 2	Model 3
Full siblings (total population)	3 153 219	1.20 (1.18 to 1.22)	1.15 (1.13 to 1.17)	1.13 (1.10 to 1.15)
Full siblings (brothers only)	750 153	1.16 (1.12 to 1.20)	1.13 (1.09 to 1.17)	1.12 (1.08 to 1.16)
Full siblings (sisters only)	822 241	1.23 (1.19 to 1.27)	1.18 (1.14 to 1.22)	1.13 (1.10 to –1.17)

1. Adjusted for asthma in relatives and diabetes in index person.

2. Adjusted for sex and age of both relative and index person and asthma in relative.

3. Adjusted for sex, age, income, education level and birth country of both relative and index person and asthma in relative.

### Sensitivity analysis

Using the extended diabetes definition also including ICD-10 E12, E13, E14 and O24, online supplemental table 1; we observed, similar to the previous analysis, increased odds of type 2 diabetes in all five cohorts—general population, men among total population, women among total population, men in the Conscription Register and women in the Medical Birth Register respectively (fully adjusted OR 1.47; 95% CI 1.45 to 1.49, OR 1.30; 95% CI 1.27 to 1.32, OR 1.63; 95% CI 1.60 to 1.66, OR 1.51; 95% CI 1.45 to 1.56 and OR 2.04; 95% CI 1.96 to 2.11) (online supplemental table 4).

Regardless of whether only the ICD-10 E11 diagnosis or the drug ATC-A10B and all oral hypoglycaemic drug subgroups were considered, the odds of type 2 diabetes remained increased (fully adjusted ORs 1.30 to 1.67), online supplemental tables 5 and 6.

## Discussion

In this population-based cross-sectional study, we found an association between current asthma and type 2 diabetes, with a 47% higher prevalence of asthma among those with type 2 diabetes. Although we could not directly account for smoking habits, this relationship sustained after adjusting for BMI, as well as other demographic and socioeconomic factors, as observed in both the male and female populations. The ORs for the female-only population were higher than those for the male-only population. Using different definitions of type 2 diabetes with an extended diabetes definition based on diagnoses from inpatient and outpatient specialist care and/or medication dispenses did not seem to change the observed associations. The familial coaggregation results suggested some shared familial risk factors explaining the association between asthma and type 2 diabetes.

### Comparison with previous studies

The relationship between asthma and type 2 diabetes is considered bidirectional.^28^

Previous studies have shown that there is an increased risk of type 2 diabetes in patients with asthma^2 29 30^ and of asthma in participants with type 2 diabetes.^1 31^ In the cross-sectional study by Black *et al*, those with asthma had a higher prevalence of type 2 diabetes.^32^ Although similar findings were observed in the longitudinal study by Mueller *et al*, the association between asthma and type 2 diabetes was attenuated after adjustment for BMI. The investigators thus concluded that obesity was an important confounder in this association.^2^ In our study, we also found that obesity was a confounder in the relationship between asthma and type 2 diabetes; however, an association still remains.

In the reverse direction, an association has also been observed between diabetes and adult-onset asthma.^31^ According to Thomsen *et al*, this association remained even after adjusting for important risk factors like smoking, age and BMI.^1^ According to Baek *et al*, despite similar findings, it was observed that only participants with severe or uncontrolled type 2 diabetes are at higher risk of developing asthma.^31^

Several mediators are possible for the association between asthma and type 2 diabetes. The regular use of oral corticosteroids is associated with older age at asthma diagnosis.^33^ Glucocorticoids are associated with a strong dose-dependent risk for obesity and type 2 diabetes^34^ and tend to be prescribed in severe asthma. Further, asthma is associated with increased production of IL-6. High levels of chronic systemic IL-6 cause insulin resistance and general metabolic dysfunction. IL-6 impairs the phosphorylation of insulin receptor and insulin receptor substrate-1 by inducing the expression of suppressor of cytokine signalling-3, a potential inhibitor of insulin signalling.^35^ This is important in the pathogenesis of type 2 diabetes.

Conversely, in the opposite direction, type 2 diabetes may also increase the likelihood of asthma. Hyperglycaemia leads to the production of advanced glycation end-products which induce oxidative stress; the resulting oxidative stress and pro-inflammatory responses cause microvascular damage and may induce dysfunction of the alveolar microvasculature.^31 36^ This may predispose patients with type 2 diabetes to asthma. In the Canadian National Population Health Survey, it was observed that obesity was related to the development of asthma in women but not in men.^37^ Obesity is associated with more severe asthma and may lead to prescribing inhaled corticosteroids more frequently or at higher doses.^38^ This may lead to a higher risk of type 2 diabetes,^39 40^ although this was disputed in a Swedish study by Hedberg and Rössner, who concluded that there was no strong evidence to suggest that modern pharmacological asthma treatment may contribute much to the development of obesity in either men or women taking asthma medication.^41^ They instead suggested that obesity was a modifiable risk factor for asthma.^41^

Furthermore, hyperinsulinaemia, associated with obesity, may inhibit surfactant A and D production by shifting T cells towards a Th2-type response, promote mast cell survival and proliferate and contract airway smooth muscle cells, all predisposing the individual to asthma.^42^ Additionally, obesity is characterised by a low-grade systemic inflammation with increased proinflammatory cytokines and adipokines.^40^

We went further to expand our findings in the familial co-aggregation where even after controlling for co-occurrence of asthma and diabetes in full siblings, we found that there are shared familial factors in this relationship. These shared familial factors could be, for example, shared genetic components or lifestyle factors. A previous study on the association between asthma and type 1 diabetes also found familial coaggregation with estimates of (OR 1.27, 95% CI 1.13 to 1.42).^43^ Although type 1 and type 2 diabetes have different aetiologies and manifestations, shared familial factors could still be similar. Future studies using other genetically informative designs will be able to elucidate how shared familial factors contribute to asthma and type 2 diabetes. For example, estimating shared heritability, linkage disequilibrium score regression, candidate genes or genome-wide cross-trait analyses will be instrumental in the understanding of the comorbidity.

### Strengths and limitations

The strengths of our study include its large size, inclusion of the total population and several confounding variables. We also used BMI to account for some residual confounding. However, we acknowledge that BMI information was only available at antenatal booking in the Medical Birth Register (for women) and at call-up in the Conscription Register (for men); the measures were thus taken several years prior to the study period for many individuals. However, it has been shown that BMI tends to be consistent over the life course.

The unavailability of dispensed prescriptions before July 2005 made it impossible to conduct a prospective study, and there would have been no way of knowing which participants had asthma in childhood. Asthma is known to have its onset in childhood, with approximately two thirds of children experiencing remission in childhood. We could not account for this in our study.

Furthermore, we could not account for those who had diabetes but had not yet filled out a prescription or been given an in-patient diagnosis. We could not ascertain that other conditions such as autoimmune diseases may not also coaggregate with asthma and type 2 diabetes as we were unable to adjust for them. While we attempted to exclude all individuals with COPD, we did not conduct a separate analysis looking at this, as analysing individuals with COPD would have necessitated a re-evaluation of the study population and required adjustments to account for differences in disease onset, confounders and thus extended the scope of our analysis.

Our findings may certainly have a clinical application. Although they could be partly due to surveillance bias due to more frequent healthcare contacts in patients with chronic illnesses, as could be the case for other studies in the field. The association between asthma and type 2 diabetes underlines the importance of screening for diabetes.

## Conclusions

We found an association between asthma and type 2 diabetes that remained significant also after adjusting for BMI. We also found that asthma and type 2 diabetes coaggregate in full siblings, indicating that the association is partly due to shared genetic and environmental risk factors. Thus, the family history of either disorder should indicate ascertainment of the other, and future studies should further investigate genetic variants underlying the co-occurrence.

## Supplementary material

10.1136/thorax-2024-222819 online supplemental file 1

10.1136/thorax-2024-222819 online supplemental file 2

## Data Availability

Data are available on reasonable request.
