# Supplementary material for: Association between asthma and type 2 diabetes in a Swedish adult population: a register-based cross-sectional study
Source: Thorax. 2025 Mar 23;80(6):e222819. doi: 10.1136/thorax-2024-222819 (PMC12128769; doi:10.1136/thorax-2024-222819)
Supplement: online supplemental file 1 [file thorax-80-6-s001.docx]

**The association between asthma and type 2 diabetes in a Swedish adult population: a register-based cross-sectional study**

*Mubanga et al.*

**Supplementary Material**

**Additional Register Information**

**Supplementary Table 1.** Summary of the diagnoses included in the *extended diabetes definition*

**Supplementary Table 2:** Baseline characteristics of the population including the *extended diabetes* cohort

**Supplementary Table 3.** Baseline characteristics of the men only and women only populations.

**Supplementary Table 4.** Odds ratios for within-person association between asthma and type 2 diabetes in general population using an *extended diabetes definition*

**Supplementary Table 5.** Odds ratios for within-person association between asthma and type 2 diabetes using a hospital only definition of type 2 diabetes from National Patient Register (ICD-10: E11)

**Supplementary Table 6.** Odds ratios for within-person association of type 2 diabetes and asthma using a definition of type 2 diabetes based on the Swedish Prescribed Drug Register.

**Supplementary Information**

Participants were identified using the Register of the Total Population with information on birth, migration and civil status on all Swedish residents.^1^ From the National Patient Register,^2^ we extracted information on physician-diagnosed asthma or type 2 diabetes. This register has had complete coverage for inpatient hospital diagnoses since 1987, and approximately 80% of outpatient specialist care visits since 2001. From the Swedish Prescribed Drug Register, available since July 2005, we were able to extract information on drugs indicated for treatment of the disease conditions of interest which have been dispensed at pharmacies in Sweden.^3^ The Longitudinal Integration Database for Health Insurance and Labor Market Studies (LISA)^4^ includes micro data about highest attained education by calendar year and income.^4^ We used it to extract information on socioeconomic status measured as education and household disposable income in 2011. The Cause of Death Register has been in existence since 1952,^5^ and was used to provide information on death dates used for exclusion. Data were linked on an individual level using the personal identity number, which is unique for each Swedish resident.^6^

**Supplementary Table 1.** Summary of the diagnoses that were included in the *extended* *diabetes* definition defined by ICD-10 codes E11, E12, E13 and E14 and O24.

| **ICD CODE** | **NUMBER** | **OVERLAP WITH E11** |
| --- | --- | --- |
| E11 type 2 diabetes | 155,396 |  |
| E12 malnutrition-related diabetes mellitus | 126 | 74 |
| E13 other specified diabetes mellitus | 795 | 501 |
| E14 unspecified diabetes mellitus | 17,424 | 11,404 |
| O24 gestational diabetes | 3,442 | 379 |

**Supplementary Table 2:** Baseline Characteristics including the *extended diabetes* cohort.*

|  | **Total Population** | **No Asthma or Diabetes** | **Asthma only** | **Diabetes only** | **Both asthma and Diabetes** |
| --- | --- | --- | --- | --- | --- |
| **Characteristics** |  |  |  |  |  |
| **Number (%)** | 5,250,870 (100) | 4,647,024 (88.5) | 336,898 (6.4) | 241,656 (4.6) | 25,292 (0.5) |
| **Sex** |  |  |  |  |  |
| *Male* | 2,588,027 (49.3) | 2,303,317 (49.6) | 131,701 (39.1) | 142,226 (58.8) | 10,783 (42.6) |
| **Age category** |  |  |  |  |  |
| *<45* | 2,145,723 (40.9) | 1,991,191 (42.9) | 131,474 (39.0) | 20,332 (8.4) | 2,726 (10.8) |
| *45 - 65* | 2,094,413 (39.9) | 1,827,758 (39.3) | 139,389 (41.4) | 114,709 (47.5) | 12,557 (49.6) |
| *>65* | 1,010,734 (19.2) | 828,075 (17.8) | 66,035 (19.6) | 106,615 (44.1) | 10.009 (39.6) |
| **Education level** |  |  |  |  |  |
| *Compulsory* | 1,055,959 (20.1) | 893,769 (19.2) | 63,612 (18.9) | 90,116 (37.3) | 8,462 (33.5) |
| *Secondary* | 2,368,249 (45.1) | 2,100,675 (45.2) | 151,918 (45.1) | 104,211 (43.1) | 11,445 (45.2) |
| *Tertiary* | 1,799,029 (34.3) | 1,629,394 (35.1) | 120,031 (35.6) | 44,513 (18.4) | 5,091 (20.1) |
| *Missing* | 27,633 (0.5) | 23,186 (0.5) | 1,337 (0.4) | 2,816 (1.2) | 294 (1.2) |
|  |  |  |  |  |  |
| **Quantiles of income** |  |  |  |  |  |
| *1 (lowest)* | 1,050,181 (20.0) | 919,015 (19.8) | 68,144 (20.2) | 56,395 (23.3) | 6,627 (26.2) |
| *2* | 1,050,303 (20.0) | 922,535 (19.8) | 70,966 (21.1) | 50,948 (21.1) | 5,854 (23.2) |
| *3* | 1,050,020 (20.0) | 927,533 (20.0) | 68,264 (20.3) | 49,201 (20.4) | 5,022 (19.9) |
| *4* | 1,050,207 (20.0) | 934,991 (20.1) | 64,453 (19.1) | 46,479 (19.2) | 4,284 (16.9) |
| *5 (highest)* | 1,050,159 (20.0) | 942,95 (20.3) | 65,071 (19.3) | 38,633 (16.0) | 3,505 (13.9) |
|  |  |  |  |  |  |
| **Country of birth** |  |  |  |  |  |
| *Sweden* | 4,544,298 (86.5) | 4,031,793 (86.8) | 295,160 (87.6) | 196,957 (81.5) | 20,388 (80.6) |
| *Other Nordic* | 162,344 (3.1) | 138,033 (3.0) | 12,080 (3.6) | 10,911 (4.5) | 1,320 (5.2) |
| *Other Countries* | 544,080 (10.4) | 477,062 (10.2) | 29,651 (8.8) | 33,784 (14.0) | 3,583 (14.2) |
| *Missing* | 148 (<0.0) | 136 (<0.0) | 7 (<0.0) | 4 (<0.0) | 1 (<0.0) |

*Describes the characteristics for total population of 5,629,066 between 2009 and 2013. Education level and quantiles of income are based on LISA data from 2011.

**Supplementary Table 3.** Baseline characteristics of the men in the Conscription Register (sub-group of men only), and the Medical Birth Register (sub-group of women only).

Baseline Characteristics of Men in Conscription Register. Describes the characteristics for the population of 1,535,814 between 2009 and 2013. Diabetes defined as ICD-10 E11 and/or ATC code - A10B.

|  | **Total** | **Neither** | **Asthma** | **Diabetes** | **Both** |
| --- | --- | --- | --- | --- | --- |
| Total | 1,535,814 (100) | 1,413,082 (92.0) | 79,286 (5.2) | 40,001 (2.6) | 3,445 (0.2) |
| **Age category** |  |  |  |  |  |
| *<45* | 950,827 (61.9) | 892,509 (63.2) | 48,609 (61.3) | 8,821 (22.0) | 888 (25.8) |
| *45 - 65* | 584,987 (38.1) | 520,573 (36.8) | 30,677 (38.9) | 31,180 (78.0) | 2,557 (74.2) |
| **Body Mass Index** |  |  |  |  |  |
| *18.5 <* | 116,071 (7.6) | 108,304 (7.7) | 5,510 (7.0) | 2,111 (5.3) | 146 (4.2) |
| *18.5 – 24.9* | 1,128,077 (73.4) | 1,047,715 (74.1) | 56,293 (71.0) | 22,247 (55.6) | 1,822 (52.9) |
| *25.0 – 29.9* | 135,538 (8.8) | 119,909 (8.5) | 7,690 (9.7) | 7,262 (18.2) | 677 (19.6) |
| *≥30* | 28,343 (1.9) | 23,853 (1.7) | 1,606 (2.0) | 2,630 (6.6) | 254 (7.4) |
| *Missing* | 127,785 (8.3) | 113,301 (8.0) | 8,187 (10.3) | 5,751 (14.3) | 546 (15.9) |
|  |  |  |  |  |  |
| **Education Level** |  |  |  |  |  |
| *Compulsory* | 208,266 (13.6) | 187,640 (13.3) | 10,023 (12.6) | 9,855 (24.6) | 748 (21.7) |
| *Secondary* | 793,333 (51.7) | 728,306 (51.5) | 41,124 (51.9) | 21,980 (55.0) | 1,923 (55.8) |
| *Tertiary* | 531,285 (34.6) | 494,440 (35.0) | 28,018 (35.3) | 8,063 (20.2) | 764 (22.3) |
| *Missing* | 2,930 (0.2) | 2,696 (0.2) | 121 (0.2) | 103 (0.3) | 10 (0.2) |

Baseline Characteristics of women in Medical Birth Register. Describes the characteristics for the population of 1,223,304 between 2009 and 2013. Diabetes defined as ICD-10 E11 and/or ATC code - A10B.

|  | **Total** | **Neither** | **Asthma** | **Diabetes** | **Both** |
| --- | --- | --- | --- | --- | --- |
| Total | 1,223,304 (100) | 1,108,035 (90.6) | 92,070 (7.5) | 19,702 (1.6) | 3,497 (0.3) |
| **Age category** |  |  |  |  |  |
| *<45* | 708,446 (57.9) | 651,314 (58.8) | 50,681 (55.1) | 5,422 (27.5) | 1,029 (29.4) |
| *45 - 65* | 510,222 (41.7) | 452,864 (40.9) | 41.007 (44.5) | 13,939 (70.8) | 2,412 (69.0) |
| *>65* | 4,636 (0.4) | 3,857 (0.3) | 382 (0.4) | 341 (1.7) | 56 (1.6) |
| **Body Mass Index** |  |  |  |  |  |
| *18.5 <* | 39,498 (3.2) | 36,511 (3.3) | 2,706 (2.9) | 246 (1.2) | 35 (1.0) |
| *18.5 – 24.9* | 722,852 (59.1) | 667,437 (60.2) | 49,156 (53.4) | 5,384 (27.3) | 875 (25.0) |
| *25.0 – 29.9* | 230,911 (18.9) | 205,532 (18.6) | 19,087 (20.7) | 5,368 (27.2) | 924 (26.4) |
| *≥30* | 88,368 (7.2) | 73,437 (6.6) | 9,315 (10.1) | 4,668 (23.7) | 948 (27.1) |
| *Missing* | 141,675 (11.6) | 125,118 (11.3) | 11,806 (12.8) | 4,036 (20.5) | 715 (20.5) |
| **Education Level** |  |  |  |  |  |
| *Compulsory* | 119,447 (9.8) | 104,815 (9.5) | 9,625 (10.5) | 4,288 (21.8) | 719 (20.6) |
| *Secondary* | 578,353 (47.3) | 522,974 (47.2) | 43,167 (46.9) | 10,335 (52.5) | 1,877 (53.7) |
| *Tertiary* | 523,866 (42.8) | 478,848 (43.2) | 39,150 (42.5) | 4,983 (25.3) | 885 (25.3) |
| *Missing* | 1,638 (0.1) | 1.398 (0.1) | 128 (0.1) | 96 (0.5) | 16 (0.4) |

**Supplementary Table 4.** Odds ratios for the within-person association between asthma and type 2 diabetes in the general population using an *extended diabetes* definition of ICD-10 E11, E12, E13 E14 and O24.

| **Population** | **N†** | **N‡** | **Model 1**  Odds Ratio  (95% CI) | **Model 2**  Odds Ratio  (95% CI) | **Model 3**  Odds Ratio  (95% CI) | **Model 4**  Odds Ratio  (95% CI) |
| --- | --- | --- | --- | --- | --- | --- |
| Total Population | 5,250,870 | 5,201,728 | 1.37 (1.36-1.39) | 1.44 (1.42-1.46) | 1.47 (1.45-1.49) | - |
| Total population  (Men only) | 2,577,583 | 2,564,529 | 1.28 (1.25-1.30) | - | 1.30 (1.27-1.32) | - |
| Total population  (Women only) | 2,651,662 | 2,637,199 | 1.60 (1.57-1.63) | - | 1.63 (1.60-1.66) | - |
| Men from Conscription Register | 1,535,814 | 1,412,700 | 1.49 (1.44-1.55) | - | 1.51 (1.45-1.56) | 1.45 (1.39-1.51) |
| Women from Medical Birth Register | 1,223,304 | 1,013,133 | 2.04 (1.97-2.12) | - | 2.04 (1.96-2.11) | 1.76 (1.68-1.84) |

**N†** Total number of people in the specified cohort

**N‡** Number of participants in fully adjusted model

1. Model 1: adjusted for age
2. Model 2: adjusted for age, sex*
3. Model 3: adjusted for age, sex*, education level, income, birth country
4. Model 4: adjusted for age, sex*, education level, income, birth country, body mass index

Sex* - this covariate is not included for the men only and female only cohorts

**Supplementary Table 5:** Using a hospital based definition of type 2 diabetes from the National Patient Register ICD-10: E11

| **Total population** | **Number with ICD-10 E11** | **Model 1** | **Model 2** | **Model 3** |
| --- | --- | --- | --- | --- |
| N | N (%) | Odds Ratio (95% CI) | Odds Ratio (95% CI) | Odds Ratio (95% CI) |
| 5,629,066 | 205,337 (3.56) | 1.54 (1.52-1.56) | 1.54 (1.52-1.56) | 1.56 (1.53-1.58) |

1. Model 1 only independent variable (asthma) and dependent variable (type 2 diabetes)
2. Model 2 adjusted for age
3. Model 3 adjusted for age, sex, education level, income, birth country

**Supplementary Table 6:** Using a definition of type 2 diabetes based on the Swedish Prescribed Drug Register.

| **Drug Category (ATC-code)** | N (%) | **Model 1**  Odds Ratio  (95% CI) | **Model 2**  Odds Ratio  (95% CI) | **Model 3**  Odds Ratio  (95% CI) |
| --- | --- | --- | --- | --- |
| All oral-hypoglycemic drugs (A10B) | 282,759 (5.0) | 1.44 (1.42-1.46) | 1.36 (1.34-1.38) | 1.46 (1.44-1.48) |
| Biguanides (A10BA) | 265,788 (4.7) | 1.44 (1.42-1.46) | 1.43 (1.41-1.46) | 1.46 (1.43-1.48) |
| Sulfonylureas (A10BB) | 77,490 (1.4) | 1.30 (1.26-1.33) | 1.28 (1.25-1.32) | 1.30 (1.27-1.34) |
| Thiazolidinediones (A10BG) | 8,594 (0.1) | 1.66 (1.54-1.78) | 1.65 (1.54-1.78) | 1.67 (1.55-1.80) |
| Combination oral hypoglycemic drugs (A10BD) | 6,576 (0.1) | 1.57 (1.44-1.71) | 1.61 (1.48-1.75) | 1.60 (1.47-1.75) |

1. Model 1 only independent variable (asthma) and dependent variable (type 2 diabetes)
2. Model 2 adjusted for age, sex
3. Model 3 adjusted for age, sex, education level, income, birth country

**References**

1. Ludvigsson JF, Almqvist C, Bonamy A-KE, et al. Registers of the Swedish total population and their use in medical research. *European journal of epidemiology* 2016;31(2):125-36.

2. Ludvigsson JF, Andersson E, Ekbom A, et al. External review and validation of the Swedish national inpatient register. *BMC public health* 2011;11(1):450.

3. Wettermark B, Hammar N, MichaelFored C, et al. The new Swedish Prescribed Drug Register—Opportunities for pharmacoepidemiological research and experience from the first six months. *Pharmacoepidemiology and Drug Safety* 2007;16(7):726-35. doi: <https://doi.org/10.1002/pds.1294>

4. Ludvigsson JF, Svedberg P, Olén O, et al. The longitudinal integrated database for health insurance and labour market studies (LISA) and its use in medical research. *European Journal of Epidemiology* 2019;34(4):423-37. doi: 10.1007/s10654-019-00511-8

5. Brooke HL, Talbäck M, Hörnblad J, et al. The Swedish cause of death register. *European journal of epidemiology* 2017;32(9):765-73. doi: 10.1007/s10654-017-0316-1 [published Online First: 2017/10/05]

6. Ludvigsson JF, Otterblad-Olausson P, Pettersson BU, et al. The Swedish personal identity number: possibilities and pitfalls in healthcare and medical research. *European Journal of Epidemiology* 2009;24(11):659-67. doi: 10.1007/s10654-009-9350-y
