# Supplementary material for: Association between asthma and type 2 diabetes in a Swedish adult population: a register-based cross-sectional study
Source: Thorax. 2025 Mar 23;80(6):e222819. doi: 10.1136/thorax-2024-222819 (PMC12128769; doi:10.1136/thorax-2024-222819)
Supplement: online supplemental file 2 [file thorax-80-6-s002.pptx]

## Slide 1
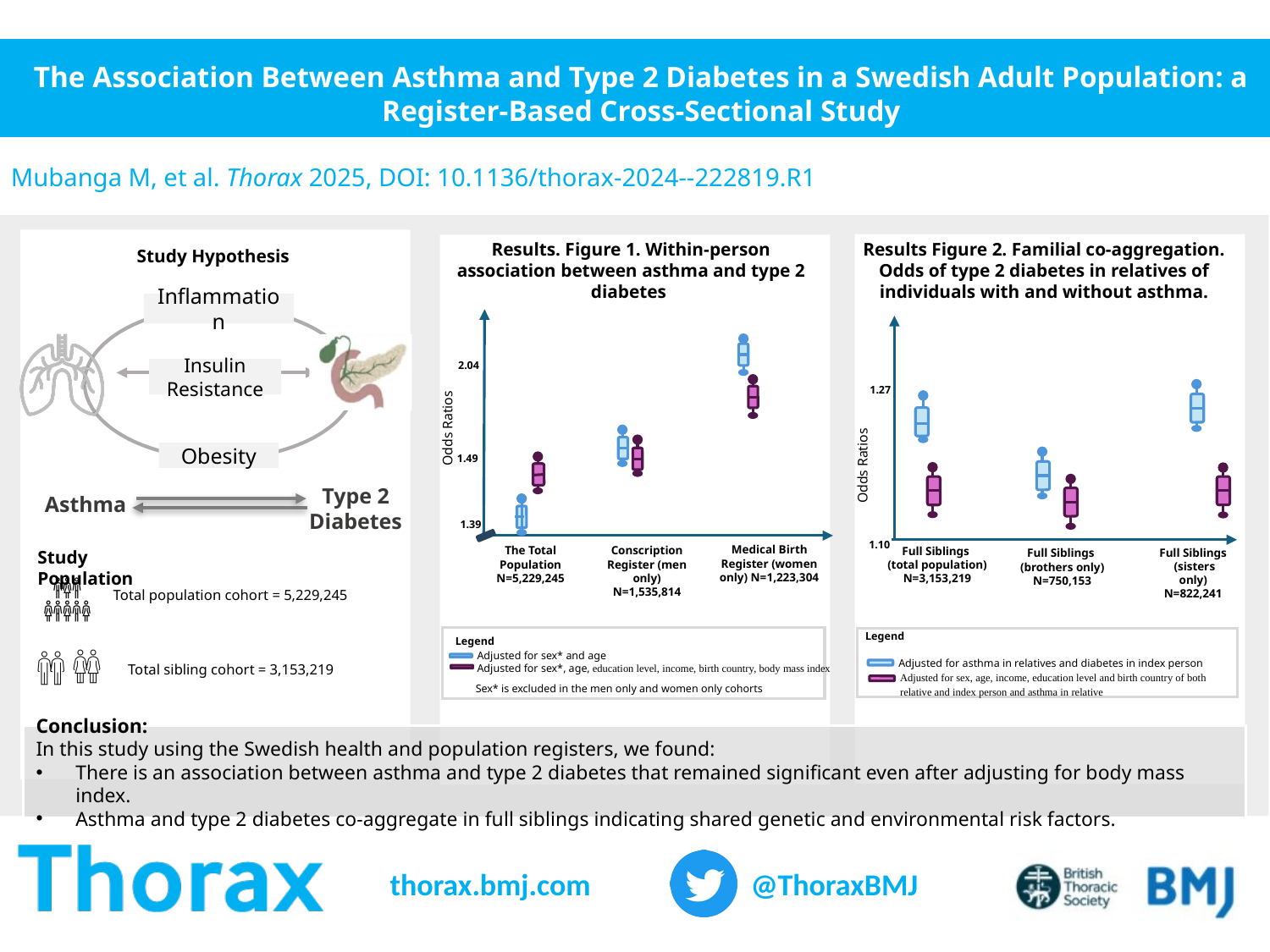

The Association Between Asthma and Type 2 Diabetes in a Swedish Adult Population: a Register-Based Cross-Sectional Study
Mubanga M, et al. Thorax 2025, DOI: 10.1136/thorax-2024--222819.R1
Results. Figure 1. Within-person association between asthma and type 2 diabetes
Results Figure 2. Familial co-aggregation. Odds of type 2 diabetes in relatives of individuals with and without asthma.
Study Hypothesis
Manuscript Title
Inflammation
2.04
Odds Ratios
1.49
Medical Birth Register (women only) N=1,223,304
The Total Population
N=5,229,245
Conscription Register (men only)
N=1,535,814
Legend
Adjusted for sex* and age
Adjusted for sex*, age, education level, income, birth country, body mass index
Sex* is excluded in the men only and women only cohorts
1.39
Full Siblings
(total population)
N=3,153,219
Full Siblings
 (sisters only)
N=822,241
Full Siblings
(brothers only)
N=750,153
Insulin Resistance
1.27
Odds Ratios
Obesity
Type 2 Diabetes
Asthma
1.10
Study Population
Total population cohort = 5,229,245
Legend
Adjusted for asthma in relatives and diabetes in index person
Total sibling cohort = 3,153,219
Adjusted for sex, age, income, education level and birth country of both relative and index person and asthma in relative
© Author(s) (or their employer(s) 2019. Re-use permitted under CC BY. Published by BMJ.
Conclusion:
In this study using the Swedish health and population registers, we found:
There is an association between asthma and type 2 diabetes that remained significant even after adjusting for body mass index.
Asthma and type 2 diabetes co-aggregate in full siblings indicating shared genetic and environmental risk factors.
thorax.bmj.com @ThoraxBMJ
